# Supplementary material for: Are carbon emissions trading and green financial instruments synergistic? -Comprehensive quantitative research based on content analysis
Source: PLoS One. 2024 Mar 7;19(3):e0298601. doi: 10.1371/journal.pone.0298601 (PMC10919601; doi:10.1371/journal.pone.0298601)
Supplement: S1 Data — (ZIP) [file pone.0298601.s001.zip › Data/地级市Z_F工作报告.zip!/╣≤╓▌/┴∙┼╠╦«╩╨/└┤╫╘╣1⁄2╓┌║┼í╛┬φ┐╦╩2╛▌═°í┐_2019.docx]

各位代表：

现在，我代表市人民政府向大会作工作报告，请予审议，并请市政协委员和其他列席人员提出意见。

一、2018年政府工作回顾

过去一年，是外部环境深刻变化的一年，也是各种矛盾交织叠加的一年，在市委的坚强领导下，在市人大、市政协的监督和支持下，我们坚持以习近平新时代中国特色社会主义思想为指导，全面贯彻党的十九大和十九届二中、三中全会精神，深入贯彻习近平总书记对贵州工作的系列重要指示批示精神，统筹推进“五位一体”总体布局，协调推进“四个全面”战略布局，坚持稳中求进工作总基调，坚持新发展理念，大力弘扬新时代贵州精神，牢牢守住三条底线，统筹推进稳增长、促改革、调结构、惠民生、防风险工作，三大攻坚战开局良好，实现经济持续健康发展和社会和谐稳定。预计地区生产总值突破1500亿元大关，增长9%左右；人均地区生产总值5.18万元，比上年增加0.17万元，比全省高1.18万元；全员劳动生产率每人9.6万元，比上年增加0.56万元，比全省高2.8万元；固定资产投资增长18%；规模以上工业增加值增长7.6%；社会消费品零售总额增长10%；一般公共预算收入增长2.24%；金融机构贷款余额增长12.8%；城镇登记失业率3.48%。

一年来，按照习近平总书记对经济高质量发展的要求，全市经济社会发展沿着“六有”方向迈进。产品有市场方面，农产品产销两旺，540吨“弥你红”红心猕猴桃远销俄罗斯、加拿大、台湾等国家和地区；规模以上工业产品产销率达到98.9%，比上年提高0.1个百分点；全年接待游客人次、旅游总收入分别增长40%和50%。投资有回报方面，亿元投资GDP产出达到1.17亿元，较上年提高0.29亿元；三次产业亿元投资投入产出分别达到1.51亿元、3.55亿元和0.66亿元，分别比上年提高0.42亿元、1.21亿元和0.17亿元。员工有收入方面，城镇新增就业9.25万人；城镇、农村居民人均可支配收入分别达到30402元、9976元，分别增长9%左右和10%左右。企业有利润方面，规模以上工业企业利润总额增长50%，每百元资产实现的主营业务收入达到52.2元、比上年提高2.1元。政府有税收方面，在严格落实国家减税降费政策、为市场主体减税17.22亿元的情况下，税收收入仍增长8.29%。环境有改善方面，森林覆盖率提高近3个百分点，达到59%；千人以上集中式饮用水水源地水质达标率稳定保持100%，单位地区生产总值能耗下降3%，主要污染物排放总量控制在省下达的指标范围内；绿色经济“四型十五种”产业占地区生产总值比重达到40%，比上年提高5个百分点。

一年来，我们按照稳就业、稳金融、稳外贸、稳外资、稳投资、稳预期的要求，保持定力、迎难而上、扎实工作，各方面都取得了新的成绩。

（一）脱贫攻坚取得决定性胜利。围绕实现“两不愁三保障”，坚持以脱贫攻坚统揽经济社会发展全局，狠抓“五个专项治理”，实施“四个聚焦”，推进“四个三”攻坚举措，“春风行动”和“夏秋攻势”尽锐出战，“秋后喜算丰收账”和“冬季充电”有力开展，“四场硬仗”再传捷报，预计减少贫困人口10.8万人，贫困发生率下降到3.74%，六枝特区和盘州市贫困发生率均下降到2%以下，脱贫摘帽工作通过市级审查，并报省评估检查。落实农业产业革命“八要素”要求，五大扶贫主导产业全面完成省下达计划，产业扶贫带动8万贫困人口增收。新建成农村“组组通”公路3914公里，实现30户以上村民组100%通水泥路。2017和2018年度易地扶贫搬迁任务全面完成，在全省率先开展易地扶贫搬迁安置点和谐社区、活力社区创建。超过33万人次的贫困学生享受到教育资助，所有深度贫困村和易地扶贫搬迁安置点卫生室规范化建设实现全覆盖。农村危房改造任务全面完成。生态扶贫走出新路，水城县入围2018年全省整县碳汇扶贫试点。东西部扶贫协作迈上新台阶，与大连市在互访交流、资金投入和项目合作等方面取得新突破，鞍钢集团、中科大、中科院定点帮扶取得新进展。

（二）污染防治实现重大突破。坚决扛起环保督察问题整改责任，中央、省委环保督察交办信访案件办结率分别为100%和95%，中央环保督察反馈意见省交办问题整改完成率达到87%。统筹推进蓝天保卫、碧水保卫、净土保卫、固废治理和乡村整治“五场战役”。市中心城区开展蓝天保卫战扬尘治理六大专项行动以来，未出现一天污染天气，全年环境空气质量优良率达到96.4%，优的天数比上年增加44天，主要污染物PM10、PM2.5平均浓度首次达到国家二级标准。地表水省控以上断面水质优良率完成省下达任务；国家下达的黑臭水体治理任务全面完成，市民对水城河治理的满意率达到98%。完成土壤详查阶段性任务，治理历史遗留重金属废渣235万吨。综合整治农村人居环境，农村垃圾、污水治理力度进一步加大，实施改厨33.83万户、改厕23.14万户、改圈10.26万户。大力推动爱绿植绿护绿，完成营造林85.54万亩，治理水土流失面积170平方公里、石漠化面积65.74平方公里；获批创建国家森林城市，娘娘山国家湿地公园顺利通过验收。启动生物多样性调查，完成野钟黑叶猴自然保护区、玉舍老林沟自然保护点总体规划编制，森林资源和野生动植物得到有效保护。

（三）防范化解风险有力有效。全面清理政府隐性债务，严控政府性债务增量。通过建立偿债风险准备金、开展政府债券置换和推进PPP模式等，化解政府性债务风险，全市未发生一起债务违约事件。加强金融运行监管，妥善处置非法集资，深入开展网络借贷风险专项整治，金融形势平稳可控。始终把安全生产作为泰山之责，对市县两级煤矿安全监管人员进行集中培训，在63家企业开展企业全员安全生产责任制试点，完成安全生产强基固本两年攻坚和遏制重特大事故试点城市年度任务，生产安全事故起数和死亡人数持续“双降”。全年未发生地质灾害伤亡。深入开展扫黑除恶专项斗争，持续深化严打整治专项行动，加强社会矛盾纠纷排查化解，有力保障了人民安居乐业、社会安定有序。

（四）产业转型升级可圈可点。坚持提升传统产业、培育新兴产业两手抓，“双百工程”实施技改工业企业148家、引进外来企业110家。启动煤炭工业转型升级高质量发展三年攻坚行动计划，释放煤炭产能840万吨/年，采煤机械化率达到82.5%、排全省第一，发耳煤矿建成西南地区第一个通过省级验收且正常生产的智能化采煤工作面。发布六盘水焦煤价格指数，填补了全省煤炭价格指数体系建设空白。纺织、新材料、新能源、旅游装备、农产品加工、物联网和现代物流七大产业板块全链条发展指导意见出台实施。电解铝全部实现就地深加工。玄武岩连续纤维产能达到1.5万吨，规模全国最大。大力发展以物联网为重点的大数据产业，与华为、科大讯飞等多家企业联合组建六盘水物联网产业联盟，我市成为中国电信物联网示范城市、中国联通智慧城市试点城市，大数据企业主营业务收入增长14%。深入实施振兴农村经济的深刻的产业革命，完成省下达的玉米调减任务，“3155工程”投产面积达152万亩。刺梨产业国家创新联盟获批成为全省第一个林业和草原国家创新联盟；发布了全省第一个市级地方标准体系《六盘水市猕猴桃生产技术标准体系》。畜牧业增加值占农林牧渔业增加值比重达到26%，比上年提高1个百分点以上。“1+10”现代农业产业园总产值增长7.76%，水城县现代农业产业园成为全省唯一一个获国家首批认定的现代农业产业园。加快发展以旅游业为重点的现代服务业，新增4个国家4A级景区、2个省级旅游度假区；全省“1+5个100工程”考核为“优”的26个景区中我市有9个，前五名我市占4个；水城县猴场乡猕猴桃产业园、钟山区梅花山国际滑雪场入选2018年农业农村部推荐的100个休闲农业和乡村旅游精品景点线路；“玩转六盘水”APP作为智慧旅游平台在全省率先上线。

（五）基础设施建设多点突破。紧盯国家和省政策导向、项目取向、资金投向，争取上级资金226.5亿元，增长12.7%。持续推进以“383”工程为重点的交通基础设施建设，预计完成投资267亿元。盘兴高铁开工建设，安六城际铁路顺利铺轨。六盘水至威宁高速公路建成通车，市中心城区72公里高速环线全面建成，高速公路出口由2个增加到4个；玉舍至普田、普定至盘州、纳雍至晴隆、六枝至安龙、织金至六枝5个高速公路项目纳入《贵州省新时代高速公路建设五年决战实施方案》，我市境内里程337公里，占全省新增项目总里程的17.5%。盘州官山机场选址获国家批复。持续推进以“181”工程为重点的水利基础设施建设，预计完成投资60.49亿元。开工建设中小型水库12座，懒龙河、观音岩、河头上3座中型水库主体完工，新增有效灌溉面积1.23万亩，解决25.74万农村人口饮水安全问题。持续推进以“131”工程为重点的信息基础设施建设，预计完成投资15亿元。建成六盘水智慧城市体验中心，实现与贵阳·贵安国家级互联网骨干直联点的高速互联，移动信号实现行政村全覆盖。持续推进市政基础设施建设，城市集中供暖工程新增供热面积181万平方米；市中心城区建成区雨污系统改造提升项目启动实施，建成后将基本实现污水全收集、全处理；地下综合管廊试点工作通过住建部、财政部现场考核。

（六）营商环境更加利企便民。严格执行中央和省进一步清理规范涉企收费的部署要求，为市场主体降费8300万元。水城经济开发区获批国家增量配电业务改革试点；组织市内24户用电企业与6家电厂开展电力直接交易，为企业节约电费2.58亿元。深入推进“放管服”改革，市直机关取消行政许可事项11项、下放3项；实施行政审批“六个一”改革，按时向社会公布审批服务便民化“六张清单”。进一步优化审批服务，工程建设项目审批办理时限缩减至法定时限的50%以下，固定资产投资项目的审批、核准、备案办理时限分别缩减到法定时限的25%、15%和30%；企业开办时间减少到8.5天以内，提前一年达到国家要求。率先在全省启动政务服务“一窗分类受理”改革，创新开展政务“集成套餐服务”，政务服务大厅事项办理“最多跑一次”达83%，“减证便民”共计取消证明材料170余类498个，我市被评为贵州省政务服务标准化建设示范市。大力开展优化营商环境集中整治行动，在全省首推由纪委监委牵头开展营商环境检查整治，做法得到省的肯定和推广。

（七）深化改革开放蹄疾步稳。深入推进176项改革重点任务，出台重点事项改革方案31个。全国农村改革试验区建设有序推进，入选农业农村部2018年度农村集体产权制度改革试点单位，“三变”改革入选全国产业扶贫十大机制创新典型。机构改革、国企改革、财税金融改革等稳步推进。市供销商贸集团挂牌成立。助力民营经济发展，268户小微企业纳入“125”培育名单、92户中小微企业纳入省“精专特新”培育名单、142户民营企业纳入“百企改造”工程，民营经济比重达到59%，比上年提高1.62个百分点。全年新增市场主体3.94万户。积极推进企业上市，建立上市企业和“三变”企业主体上市（挂牌）后备资源库，盘州市久泰邦达能源控股有限公司在香港上市，是贵州省第二家、我市首家上市的民营煤炭企业。全省第一家在欧洲挂牌设立驻欧洲投资促进代表处。成功举办物联网高峰论坛；妥乐论坛被列入全省推动企业沿着“一带一路”方向“走出去”行动计划，与生态文明论坛、中国—东盟教育交流周、数博会等一并作为省面向“一带一路”沿线国家举办重要经贸活动的依托平台。新引进500强企业5家。新增高新技术企业19家，首次获得中国专利奖，六盘水高新技术产业开发区获省批复，全市综合科技进步水平指数达到54%，比全省平均水平高5.4个百分点。

（八）民生各项事业全面进步。财政民生类重点支出达到231.98亿元，占财政总支出的72.84%。“十件民生实事”全面完成。全面推进基础教育领域综合改革，在全省率先对城镇低收入困难家庭子女就学进行资助、率先开展公办学校教师员额制管理改革试点、率先落实教育综合改革经费保障。启动基础教育三年攻坚，市中心城区超大班额全部消除，完成义务教育学校划片招生，小学入学年龄从6.5岁调整为6岁。提前两年实现县域义务教育基本均衡发展。六盘水幼儿师范高等专科学校挂牌招生，六盘水师范学院二期工程全面建成。14家二级以上医院纳入跨省异地就医结算系统，率先在全省开展新农合付费支付方式改革；我市获国家精神卫生综合管理示范区称号。社会保险扩面工作稳步推进，覆盖率超过94%，城乡低保、居民基本医保政府补助标准进一步提高，被财政部、民政部确定为第三批中央财政支持开展居家和社区养老服务改革试点。2018年度保障性安居工程考核成绩位列全省第一。着力丰富群众文化体育生活，获批创建国家第四批公共文化服务体系示范区；突出消夏文化节的群众性，打造“开放的平台，百姓的舞台”；获得2022年第十一届省运动会承办权，省第十届运动会参赛成绩较上届提升2位，圆满举办市第二届运动会及马拉松、滑雪、滑翔伞等系列重大赛事，入选全国青少年校园足球改革试验区。成功创建省食品安全示范城市。司法行政、民族、宗教、外事、侨务、对台、人防、档案、妇女、儿童、老龄、残疾人、地方志、红十字等各项事业取得新成绩。

各位代表！新时代对政府工作提出了新的更高要求。一年来，我们树牢“四个意识”，坚定“四个自信”，坚决做到“两个维护”，始终在政治立场、政治方向、政治原则、政治道路上同以习近平同志为核心的党中央保持高度一致。围绕信念过硬、政治过硬、责任过硬、能力过硬、作风过硬，践行“五步工作法”，不断提高施政能力和服务水平。自觉把政府工作置于市委领导之下，重要情况主动向市委请示汇报，重大问题及时提请市委研究决策。按规定向人大报告有关工作、与政协委员开展民主协商，高质量办理建议和提案，办复率和满意率均达100%。切实履行全面从严治党主体责任，严格执行中央八项规定及其实施细则精神和省委、市委作风建设有关规定，持之以恒兴“三风”、纠“四风”，巡视问题整改取得阶段性成效，党风廉政建设不断加强。

各位代表！过去一年，我们走过的路很不寻常，面对资金、土地、劳动力等要素约束趋紧，面对安全生产、环境保护、债务化解等风险防范巨大压力，我们积极应对，全市改革发展稳定局面持续向好，成绩来之不易。这是以习近平同志为核心的党中央掌舵领航、对贵州亲切关怀的结果，是省委、省政府坚强领导和关心支持的结果，是在市委领导下，全市各族干部群众艰苦奋斗、苦干实干的结果。在此，我代表市人民政府，向全市各族人民致以崇高的敬意！向人大代表、政协委员，向各民主党派和各人民团体，向驻市人民解放军、武警官兵和人民警察，向所有关心六盘水建设发展的各界人士，表示衷心的感谢！

在肯定成绩的同时，我们也清醒认识到发展不平衡不充分的问题还比较突出。目前全市还有贫困人口约9.5万人，贫困人口较多，内生动力不足，产业扶贫基础还需夯实，深度贫困地区生产生活条件还需改善，脱贫攻坚任务还十分繁重。新的经济增长点支撑作用不强，产业之间、产业内部结构性矛盾突出，结构调整面临不少困难，转型升级任务还十分艰巨。金融机构存款余额同比下降，融资难融资贵问题较为突出，地方政府性债务风险隐患不容忽视，生态环境保护任务压头，安全生产形势依然严峻。教育、卫生等公共服务供给不足、质量不高，与人民群众日益增长的美好生活需要还有较大差距。我们要全面客观准确把握形势，培养战略思维、树立底线思维，从最坏处着想、向最好处努力，居安思危、化危为安，把工作的主动权牢牢握在手上。

二、2019年政府工作主要任务

今年是新中国成立70周年和西部大开发20周年，是全面建成小康社会关键之年，做好经济社会发展各项工作至关重要、意义重大。今年政府工作的总体要求是：以习近平新时代中国特色社会主义思想为指导，全面贯彻党的十九大和十九届二中、三中全会精神，深入落实习近平总书记对贵州工作的重要指示批示精神，按照中央和全省经济工作会议决策部署，牢记嘱托、感恩奋进，统筹推进“五位一体”总体布局，协调推进“四个全面”战略布局，坚持稳中求进工作总基调，坚持新发展理念，坚持推进高质量发展，坚持以供给侧结构性改革为主线，坚持深化市场化改革、扩大高水平开放，坚持“巩固、增强、提升、畅通”八字方针，加快建设现代化经济体系，统筹推进稳增长、促改革、调结构、惠民生、防风险工作，保持经济运行在合理区间，进一步稳就业、稳金融、稳外贸、稳外资、稳投资、稳预期，提振市场信心，增强人民群众获得感、幸福感、安全感，继续打好三大攻坚战，持续推动三大战略行动，牢牢守好三条底线，决战脱贫攻坚、决胜同步小康，保持经济持续健康发展和社会大局稳定，为全面建成小康社会收官打下决定性基础，以优异的成绩庆祝中华人民共和国成立70周年！

根据市委安排，今年经济社会发展的主要预期目标是：地区生产总值增长10%左右，固定资产投资增长14%，社会消费品零售总额增长11%，金融机构存、贷款余额分别增长11%和17%，一般公共预算收入增长1%，城镇、农村居民人均可支配收入分别增长9%左右和10%左右，城镇登记失业率控制在4.2%以内，森林覆盖率达到61%以上，完成省下达的节能减排降碳任务。

围绕实现全年主要目标任务，重点抓好以下八个方面工作。

（一）全力夺取脱贫攻坚决战之年根本性胜利。巩固提升脱贫攻坚成果，坚持标准，一鼓作气，着力解决好实现“两不愁三保障”面临的突出问题，减少农村贫困人口5.2万人，贫困发生率降到3%以内。深入推进振兴农村经济的深刻的产业革命，继续调减玉米种植面积，“3155工程”投产面积165万亩；深入细致做好新增10万亩猕猴桃和100万亩刺梨的种植工作，落实地块、完善规划、创造条件，分年度积极有序推进。稳定烤烟种植面积。按照“一坝一策”原则，围绕把500亩以上坝区培育成为农业现代化的样板田、科技田、效益田，以特色化、规模化、标准化、品牌化、绿色化为方向，大力推进坝区结构调整，将10个以上坝区打造成农业产业优化升级示范区。把坝区农业结构调整与农业园区建设紧密结合起来，持续推进“1+10”现代农业产业园建设，积极推进盘州市刺梨现代农业产业园申报国家现代农业产业园。大力发展山地草食生态畜牧业，实施“粮改饲”2万亩以上，稳定发展生猪产业，加快发展种草养牛养羊，畜牧业增加值占比提高1个百分点。多措并举严防非洲猪瘟。完成“十三五”易地扶贫搬迁任务，全面推开和谐社区、活力社区创建，促进搬迁群众市民化。全面落实教育扶贫政策，确保教育扶贫资助兑现率100%、义务教育贫困学生入学率100%。加强精准健康扶贫，建档立卡贫困人口100%参合或参保。继续实施农村危房改造，按照“四个一批”要求，完成村庄老旧房整治。加快补齐以交通、水利为重点的基础设施短板，加快提升农村公路养护和管理水平，巩固“四好农村路”钟山区国家级示范县、盘州市省级示范县创建成果，支持六枝特区创建省级示范县。全面解决30户以上自然村寨饮水安全问题。大力开展扶贫扶志行动，增强贫困群众内生动力和自主脱贫能力。统筹用好各方帮扶资源，扎实推进东西部扶贫协作、定点扶贫和社会帮扶。扎实开展漏评、错评、错退专项整治，确保“三率一度”达到国家标准。用好扶贫专项，加大扶贫资金审计力度，从严从快查处贪污挪用、截留私分扶贫资金等行为。坚持严管与厚爱相结合，关心爱护基层扶贫干部。

（二）加快传统产业改造提升和新兴产业集群发展。巩固提升传统产业战略性基础地位，稳住煤电钢材四大支柱产业基本面，稳住全市工业经济基本盘。坚持“保安全、促增产”，实施煤炭产能释放三年攻坚行动，技改、建成和复工复产煤矿26处，释放煤炭产能1160万吨/年。启动10处煤矿智能机械化改造，采煤机械化率达到88%以上。加大正常生产和建设煤矿涉法涉诉、矿界重叠等问题处置力度，促进现成产能释放。积极争取省能源结构调整基金和投贷联动资金，优先支持扩产能、增产量见效快、见效大的企业，优先支持动力煤煤矿建设。淘汰煤炭落后产能120万吨/年以上。全力支持盘江煤电集团聚焦主业做大做强，全力支持首钢水钢集团做精钢铁主业、做大非钢产业，全面完成省下达的燃煤机组超低排放改造任务，积极扩大水泥生产等企业绿色工厂试点。

坚持依托资源、依据市场、依靠创新、依赖环境的基本原则，按照板块布局、产业耦合、链条发展、集聚建设的要求，一体聚焦、同向发力，稳扎稳打推进七大产业板块全链条发展。大力发展纺织产业，在确保环保达标的前提下，启动发耳织染科技产业园24万吨/年印染项目一期工程，积极推进盘北经济开发区伟亿纺织年产20万锭棉纱项目，逐步形成纺纱—织布—印染—服装加工产业链。大力发展新材料产业，稳定玄武岩连续纤维质量，加大下游产品研发生产和市场推广；推动水城经济开发区电解铝技术改造和铝产业链进一步向下延伸，推动盘北经济开发区发展铝深加工。大力发展新能源产业，加快实施10万吨高纯硫酸锰项目和1.5万吨电池级锂盐项目，力争在正极材料领域取得突破；积极开展废氢收集利用，启动液氢工厂建设；继续推进煤层气综合开发利用，煤层气抽采利用率达到47%。大力发展旅游装备产业，建成与多贝玛亚索道项目配套的热镀锌项目，启动2条索道建设；加快六盘水高新技术产业开发区房车生产项目实施；利用纺织、玄武岩连续纤维、铝材等方面的资源配套，积极发展其他旅游装备产业。大力发展农产品加工产业，继续支持人民小酒、盘县火腿发展壮大，积极开拓猕猴桃酒市场，全力突破刺梨加工和市场推广。大力发展物联网产业，扩大在工业制造、农业生产、节能环保、商贸流通等领域的应用，依托煤矿智能机械化、智慧城市建设等方面的需求，大力发展矿山传感器、电子元器件、通信模组、智能终端等产业，加快形成安防产业链。大力发展现代物流产业，实施冷链物流业三年攻坚行动，深挖公路和铁路运输潜力，推动多式联运，谋划建设现代物流中心，加快形成现代物流体系。多渠道加大七大产业板块发展投入，大力争取省绿色产业扶贫投资基金、工业及省属国有企业绿色发展基金更多支持。深入推进“百企改造”和“百企引进”。

（三）加强污染防治和生态建设。把环保督察问题整改作为重中之重，确保反馈问题按时整改到位。继续打好污染防治攻坚战，巩固扩大市中心城区蓝天保卫战扬尘治理六大专项行动成果，完成以气代煤压减燃煤污染三年行动任务，市中心城区环境空气质量优良率达到省的要求。集中力量攻坚水污染治理，严格落实“水十条”和河长制，强化集中式饮用水水源地保护，千人以上集中式饮用水水源地水质达标率保持100%，力争地表水省控以上断面水质优良率达到100%。大力推进城镇污水处理设施建设三年行动，市中心城区生活污水处理率达到90%、县城达到80%、建制镇达到35%。继续打好净土保卫、固废治理战役，完成土壤污染成因调查和地块污染风险筛查，加大重金属历史遗留废渣治理力度，提高固废综合利用率。编制完善生态保护红线、环境质量底线、资源利用上线、环境准入“负面清单”，加快建立“三线一单”管理机制。深入推进“六个一律”和“守护多彩贵州·严打环境犯罪”专项行动，严惩各类环境违法犯罪。

实施国土绿化行动和森林扩面提质增效三年行动计划，积极推进乌蒙山片区山水林田湖草生态保护修复试点工程，完成营造林72万亩，治理水土流失面积170平方公里、石漠化面积62平方公里。加大生物多样性保护力度，开展野钟黑叶猴市级自然保护区综合科学考察，对珍稀濒危野生动植物进行拯救性保护，提升生态系统抵抗和自我修复能力。坚持生态产业化、产业生态化，大力发展绿色经济“四型十五种”产业，走绿色低碳循环发展之路。

（四）大力推动城乡协同发展。坚持把城市作为城乡协同发展的龙头，加快建设百姓富、生态美，高颜值、高气质、高品质的新型现代化山地城市，常住人口城镇化率提高1.5个百分点。注重规划引领，优化空间布局，加快鱼塘片区路网和用地整理研究，推进麒麟北路及延伸段、鱼塘西路、鱼塘北路地下综合管廊建设。继续推进乌蒙大道建设，建成凤凰东路、凤凰南路，完成钢城大道二期下穿铁路工程，打通城市“断头路”。以市中心城区建成区雨污系统改造提升项目为抓手，改造疏通污水收集管网，加大污染较重河段治理力度，着力解决雨污混流问题。大力开展城镇生活垃圾处理设施建设三年行动，建设钟山区100吨/日餐厨垃圾处理设施。继续推进火车站、德坞、官寨、塔山等棚户区改造，建成城镇保障性安居工程1.37万户。启动老旧房屋电梯加装工作，改造提升生鲜店、便利店，提升物业管理服务水平，解决好人民群众衣食住行和柴米油盐酱醋茶等具体而微的事情。稳步扩大市中心城区集中供暖覆盖面，打造夏季凉爽、冬季温暖的城市。继续抓好城市绿化和美化，大力开展国家森林城市创建，全市城市建成区绿地率达到33.8%。推进六枝特区、盘州市县城改造提升，因地制宜打造各具特色的康养小镇、旅游小镇、商贸小镇、体育小镇。深入开展文明城市创建，深度挖掘和弘扬“盘县会议”红色革命精神、“三线”建设创业奉献精神、“三变”改革创新争先精神，彰显城市文化，提振市民精气神。

深入实施乡村振兴战略，强化农业农村优先发展的政策导向，突出抓好“三农”工作各项硬任务的落实。在推进农村产业革命、加快产业兴旺的同时，加快乡村人才振兴、文化振兴、生态振兴、组织振兴。建立完善人才下乡、返乡、回乡就业创业激励引导机制，实施新型职业农民培育工程，引进30名农业技术专家、培养300名农村致富带头人、培育3000名新型职业农民。加强农村公共文化建设，农村基层综合文化服务中心覆盖70%的行政村。坚持改善人居环境同农村经济发展水平相适应、同当地文化和风土人情相协调，大力实施农村人居环境整治三年行动，抓实农村厕所革命、农村生活垃圾治理、农村生活污水治理、村庄清洁四个专项行动，推广生活垃圾“户分类、村收集、镇转运、县处理”模式，科学合理利用城镇污水管网收集、建设分散式污水处理设施等处理农村污水，有序解决农村垃圾围村和污水横流问题。以“四在农家·美丽乡村”小康升级行动计划为抓手，提升村容村貌，实现公共厕所、公共照明分别覆盖75%和70%的行政村。积极开展“践行新时代村规民约示范村”创建，打造充满活力、和谐有序的善治乡村。

（五）大力推动扩投资促消费。紧盯国家保持基础设施领域补短板力度九大领域，加快推进237个重大基础设施和民生领域项目前期工作，力争60%以上项目开工建设。实施重大工程和重点项目630个，完成投资1100亿元以上。加快“三级一千”民间投资重点项目和PPP“样板工程”项目建设，带动民间投资500亿元以上。稳定以交通、水利、信息为重点的基础设施投资，分别完成交通投资310亿元、水利投资40亿元和信息投资12亿元。积极推动六威昭城际铁路和水盘城际铁路前期工作，大力推进盘兴高铁建设，基本建成安六城际铁路、启动沿线站房建设。加快赫章至六盘水高速公路建设，开工建设纳雍至晴隆、六枝至安龙高速公路，力争开工建设玉舍至普田高速公路，争取普定至盘州高速公路纳入国家高速公路网规划；开工建设六枝大用至落别一级公路。开工建设盘州官山机场试验段和盘州通用机场。深入推进水利建设“三大会战”，加快英武大型水库前期工作并力争开工，开工建设1座中型水库和5座小型水库，启动4个中小河流治理工程。基本建成信息“131”工程，实现公共WIFI县城全覆盖。促进农村电网改造升级，农村电网供电可靠率、综合电压合格率提前一年达到国家要求。

加快发展现代服务业，促进消费提质升级，增强消费对经济发展的基础性作用。实施服务业创新发展十大工程，新增1个省级服务业集聚区，新增限上批零住餐企业20家以上。用好“一江、一泉、一雪、两口气”资源，做足“天、地、人、和”文章，大力发展全域旅游，把旅游业打造成老工业城市转型发展的重要支撑，接待游客人次、旅游总收入分别增长22%和30%以上。围绕全省旅游“1+5个100工程”建设，完善明码标价、景区标识、道路导航、旅游厕所等景区设施设备，提升景区服务质量，打造更多“优”“良”级景区。启动4个重点景区提级改造，新增国家4A级景区或省级旅游度假区2个以上，推进野玉海国家级旅游度假区申报创建。支持和规范民宿发展，增加住宿供给。深入推进文旅、体旅、农旅、工旅以及旅游和康养融合发展，打造南方重要的旅居目的地。继续办好全市旅游文化产业发展大会，精心筹办2020年第十五届全省旅游产业发展大会。继续推进国家级电子商务进农村综合示范县建设，全市网络零售额增长20%以上。支持社会力量扩大教育、育幼、养老、医疗、文化、体育等服务供给，积极发展科技信息服务业。实施质量提升行动，加强消费者权益保护，维护安全放心的消费环境。

（六）坚定不移推进改革开放。积极推进正在开展的33项国家和省改革试点任务。聚焦全国农村改革试验区建设，深化农村集体产权制度改革，逐步建立规范高效的“三权分置”运行机制，不断提升“三变”改革经营主体质量效益，优化利益联结机制，强化入股农户合同书、股权证、分红单“三个到户”，打造“三变”改革升级版，使之真正成为脱贫致富的黄金腰带、现代农业的制度基础、工商资本的下乡通道、乡村善治的有效激励。深入推进“放管服”改革，对市级实施的184项行政许可事项继续清理下放；探索开展权力清单内权力行使情况报告机制，推动政府部门按清单履职尽责。积极推进投融资体制改革，抓好“先建后验”改革试点，加大企业上市培育和支持力度。完成机构改革，统筹推进财税金融、收入分配、社会保障、国有企业等领域改革。加快融入“一带一路”和长江经济带，进一步发挥好妥乐论坛、物联网高峰论坛等对外交流平台作用。强力推进产业大招商，立足现有企业和产业，加强领导招商、小分队招商、第三方平台招商，充分发挥六盘水驻欧洲投资促进代表处作用，用好招商引资奖励政策，加大上下游产业引进力度，推动补链强链延链。建好六盘水高新技术产业开发区，加快国家小型微型企业创业创新示范基地建设，打造6个市级以上科技创新平台，新增高新技术企业10家，综合科技进步水平指数提高3个百分点。

持续优化营商环境，大力支持民营企业发展壮大。坚决落实减税降费政策和降成本措施。进一步减少行政审批，企业开办时间压减至3个工作日以内，工程建设项目审批服务时间压减至80个工作日以内，电力用户办电平均时间压减至40个工作日以内。加快推进增量配电业务改革试点，积极推进电力直接交易，进一步降低企业用电成本。严格落实“六条政策措施”，实施六大专项行动，用好“服务民营企业市长直通车”，切实为民营经济营造更好发展环境，帮助民营经济解决发展中的困难，让民营经济创新源泉充分涌流，让民营经济创造活力充分迸发。设立创业投资小微企业基金，继续用好“3个15万元”政策，加大小微企业培育力度，推动大众创业万众创新。承办好第六届全球贵商发展大会。大力构建“亲”“清”新型政商关系，打造服务和效率高于周边、成本和负担低于周边的营商环境新高地。

（七）严密防范化解重大风险。严格落实“七严禁”和“1+8”债务管理系列政策，切实履行政府性债务“借用还”主体责任，综合采取推进PPP模式、盘活闲置土地等存量资产、用好偿债风险准备金和债务风险应急周转资金池等措施化解到期债务，确保不发生一起债务违约事件。开展隐性债务再核查，严控政府性债务增量。紧盯中央增加地方一般性转移支付、增发地方政府专项债券等政策措施，积极向上对接，争取得到更多支持。坚持在发展中化解矛盾、防范风险，平衡好稳增长与防风险的关系，在严控债务增量的基础上，采取市场的办法、借助市场的力量，吸引有实力的企业参与项目建设、资源开发，着力涵养和培植财源，做大财政收入总量，用发展的办法创造新财富、增强偿债能力。同时，压缩一般性支出，推进预算和绩效管理一体化，提高财政资源配置效率和使用效益。立足增强自身“造血”功能，加快平台公司实体化、市场化转型，提高自主还款能力。加强金融生态环境建设，深入开展非法集资、交易场所和互联网金融专项整治行动，加大不良贷款处置力度，坚决防范系统性风险。

（八）全力保障和改善民生。坚持把稳就业摆在突出位置，切实解决好高校毕业生、农民工、退役军人等群体就业，加强对零就业家庭成员、大龄失业人员和化解过剩产能职工等的就业帮扶，城镇新增就业9万人以上。坚持教育优先发展，大力推进基础教育改革发展追赶跨越三年攻坚，深入推进基础教育领域综合改革，学前三年毛入园率、普惠性幼儿园覆盖率、九年义务教育巩固率、高中阶段毛入学率分别达92%、80%、97%和94%以上。继续支持六盘水师范学院开展应用型高校转型试点，深化职业教育办学体制改革和育人机制改革，鼓励社会力量兴办教育，全面提高各级各类学校教育教学质量。加快“健康六盘水”建设，实施医疗卫生服务能力提升八大工程和“银龄计划”，80%的乡镇卫生院或社区卫生服务中心建成中医馆；全面推进分级诊疗，深化公立医院综合改革，县域内就诊率达到90%以上。强化社会保障兜底，社会保险覆盖率达到94.6%以上，稳步提高各类保障水平。加强农村“三留守”人员关爱保护，扎实做好城镇困难职工解困脱困工作。推进国家公共文化服务体系示范区创建，“广电云”户户用基本实现全覆盖。新建、改扩建村（居）综合文化服务中心298个。积极筹备省第十一届运动会，精心打造“一县一特、四季四品”体育赛事，广泛开展多样性群众体育活动。弘扬科技精神，普及科技知识，提高公民科学素质。进一步完善安全生产责任体系，全面推开企业全员安全生产责任制，深入开展煤矿瓦斯灾害防治攻坚、危险化学品和电气火灾综合治理等专项行动，统筹抓好道路交通、建筑施工、非煤矿山、特种设备等领域安全生产，防范和遏制重特大事故。加强食品药品安全管理，推进国家食品安全示范城市创建。加强应急能力建设，加大地质灾害防治力度，提高防灾减灾救灾能力。完善矛盾纠纷多元化解体制机制和社会治安防控体系，纵深推进扫黑除恶专项斗争，不断提升群众安全感满意度。重视国防动员、双拥共建和后备力量建设，积极支持军队、武警部队建设和监委、法院、检察院工作。加强国家安全、司法行政和民族、宗教、外事、侨务、对台、人防、档案、妇女、儿童、老龄、残疾人、地方志、红十字等工作。

继续办好“十件民生实事”。（1）新建、改扩建中小学校及幼儿园20所；（2）新建易地扶贫搬迁安置点医疗卫生项目10个、教育项目13个；（3）新增市中心城区新建小区、老旧小区供热面积100万平方米；（4）调整延长市中心城区夜间人流量较大的10条公交车线路运营服务时间；（5）新建、改建农村户用卫生厕所39000户；（6）解决13.32万农村人口饮水安全问题；（7）新建、改造农村电网中低压线路6000公里，新增配电变压器1700台、容量22万千伏安；（8）实施农村公路生命安全防护工程，处置隐患里程200公里；（9）改造城乡老年院60所，其中敬老院3所、日间照料中心12所、幸福院45所；（10）实施贫困劳动力全员培训25600人。

三、加强政府自身建设

面对艰巨繁重的改革发展任务，我们将围绕“五个过硬”要求，全面加强政府自身建设，提高施政能力和水平，在重要战略机遇期实现更大的新作为。

（一）坚定不移拥护看齐核心。坚持把政治建设摆在首位，牢固树立“四个意识”，坚定“四个自信”，做到“四个服从”，坚决维护习近平总书记党中央的核心、全党的核心地位，坚决维护党中央权威和集中统一领导，坚决杜绝“七个有之”，坚决做到“五个必须”。以求真务实作风坚决把中央和省决策部署落到实处，做到令行禁止，对党绝对忠诚。自觉强化政治责任，保持政治定力，把准政治方向，提高政治能力，增强斗争精神，坚决同破坏政治纪律和政治规矩的行为作斗争。

（二）不断加强法治政府建设。坚持法定职责必须为、法无授权不可为，带头尊崇和执行宪法，把政府工作全面纳入法治轨道。进一步加强政府立法工作，严格执行《六盘水市人民政府工作规则》，健全依法决策机制，加强合法性审查，规范决策程序。推进执法规范化建设，抓好“双随机、一公开”，对新业态新模式包容审慎监管，留足发展空间。依法接受市人大及其常委会的法律和工作监督，自觉接受市政协的民主监督，全面接受监察监督，主动接受新闻媒体和社会各界监督，认真听取人大代表、政协委员和民主党派、工商联、无党派人士、人民团体的意见。全面推进政务公开，让施政更加透明高效。

（三）自觉做到勤勉务实为民。坚持政府的所有工作都要体现人民意愿，干得好不好要看实际效果、最终由人民来评判。不驰于空想、不骛于虚声，严禁弄虚作假、虚报浮夸，杜绝“形象工程”“政绩工程”，确保工作经得起人民和历史的检验。聚焦“五个为”和“六个一”，进一步破除各种束缚创新发展活力的桎梏，让企业和群众创业创新的热情持久不衰，靠公平竞争加快实现高质量发展。大力推进“互联网+”，让百姓少跑腿、数据多跑路，不断提升公共服务均等化、普惠化、便捷化水平。坚持“三个区分开来”，褒奖和鼓励政治坚定、奋发有为的干部，警醒和惩戒慢作为、不作为、乱作为的干部。规范督查检查考核，压缩总量和频次，减少材料表格报送，把精力集中到抓落实上。

（四）持之以恒提升工作能力。坚持用习近平新时代中国特色社会主义思想武装头脑、指导实践、推动工作，及时学习领会习近平总书记最新重要讲话精神，继续在真正学懂弄通做实上下功夫。着力观大势谋大局，不断增强抢抓机遇意识，不断提升驾驭复杂局面的能力。坚持“重点论”和“两点论”相统一，弹好“钢琴”、转好“盘子”，把牢工作主动权。按照既要政治过硬、也要本领高强的要求，提升“八个本领”，让干部职工不仅有担当的宽肩膀，还有成事的真本领。

（五）落实全面从严治党责任。始终敬仰党的事业、敬畏手中的权力、敬重服务的人民，认真履行党风廉政建设主体责任和“一岗双责”，纵深推进政府系统党风廉政建设。坚决扛起巡视整改政治责任，以整改诠释忠诚。严格执行中央八项规定及其实施细则精神和省委、市委作风建设有关规定，大兴“三风”，整治“四风”。严格执行中国共产党廉洁自律准则，坚决守住理想信念高线、筑牢思想道德防线、管住从政行为底线，坚决打掉权力寻租空间、铲除腐败滋生土壤。推动全面从严治党向基层延伸，让群众感受到清正干部、清廉政府、清明政治就在身边、就在眼前。

各位代表，新时代是奋斗者的时代，新时代也是干出来的。让我们紧密团结在以习近平同志为核心的党中央周围，在市委的坚强领导下，不忘初心、牢记使命，不负嘱托、感恩奋进，用苦干实干决战脱贫攻坚、决胜同步小康，用决战决胜续写百姓富、生态美的多彩贵州新未来六盘水篇章，以优异的成绩向中华人民共和国成立70周年献礼！
